# Demographic and Clinicopathological Analysis of Leiomyosarcoma at Various Anatomical Sites: A Tertiary Care Hospital Experience

**DOI:** 10.7759/cureus.85124

**Published:** 2025-05-31

**Authors:** Jagadheeswari Sengottaiyan, Archana Balasubramanian, Barathi Gunabooshanam

**Affiliations:** 1 Pathology, Sri Ramachandra Institute of Higher Education and Research, Chennai, IND

**Keywords:** differentiation, extrauterine, fnclcc grade, leiomyosarcoma, mesenchymal, metastatic, sarcoma, smooth muscle, soft tissue, uterine

## Abstract

Introduction

Leiomyosarcoma (LMS) is a malignant tumor of smooth muscle origin, most prevalent in soft tissues and the gynecologic tract. The molecular alteration associated with this condition is genomic instability, leading to complex genotypes.

Aim

This study aimed to study the demographic and histopathological profile of LMS cases (80 cases) diagnosed in our hospital over seven years.

Materials and methods

This is a retrospective study of histologically proven LMS cases from January 2017 to December 2023 using the laboratory information system from our institution. The variables to be studied included clinical demographic data and histopathological findings.

Results

Out of 80 cases studied, 45 were resected specimens, 25 were biopsies, and 10 were referral cases. Fifty-eight out of 80 cases were primary LMS, 17 were metastases from various sites, and five were recurrent lesions. The most common site of primary lesion was the uterus, with 26% (n=15), followed by the retroperitoneum, with 21% (n=12), and the thigh, with 12% (n=7). Infrequent sites included the prostate, intestine, omentum, kidney, and testis, each constituting about 2% (n=1), with a male:female ratio of 1:1.7 and a mean age of presentation of 55 years. The average size of the excised tumor ranged from 5.5 cm to 15 cm, and around 73% presented with histological grade 2. Almost all the cases tested for immunohistochemical (IHC) expression of smooth muscle actin (SMA) and desmin showed positivity, along with a median Ki-67 labeling index of 30%. Metastatic lesions were found primarily in the liver, lungs, and soft tissues and rarely in bone.

Conclusion

The results of our study witnessed the incidence of LMS at diverse anatomical locations and dimensions along with advanced grade at presentation that determined the overall prognosis, though uterine LMS tend to be more aggressive and are prone to metastasize. Hence, the comprehensive assessment at the time of diagnosis, along with close follow-up of the patients, is recommended for early detection and management of metastatic lesions.

## Introduction

A sarcoma is a malignant tumor originating from mesenchymal cells that are derived from the mesodermal germ layer during embryonic development, which gives rise to connective tissue, muscle, bone, and vascular structure. Two distinct muscle types in our body include skeletal muscles, which are attached to the bones and tendons and are responsible for locomotion and posture, along with smooth muscles, which are found in the walls of hollow organs, muscles of the skin, erectile muscles of the nipple and scrotum, and iris of the eye and are responsible for their function [1]. Tumors arising from the latter are called leiomyomas, which are benign entities, and leiomyosarcomas (LMS) are malignant counterparts [2]. The female genital tract, especially the uterus, is the most common site of occurrence of these two tumors, followed by the gastrointestinal tract, retroperitoneum, trunk and extremities, head and neck, orbit, inferior vena cava (IVC), urinary bladder, and thoracic organs [2].

Microscopically, they both contain bundles and fascicles of spindle-shaped smooth muscle cells; nevertheless, the presence of atypia, increased mitosis, and necrosis is the defining feature of LMS [2]. Based on the degree of differentiation, extrauterine LMS can be scored as one, two, and three for well, moderately, and poorly differentiated tumors, respectively, along with the percentage of necrosis and number of mitoses per mm² that determine the grading of this sarcoma according to the French Federation of Cancer Centers Sarcoma Group (FNCLCC) grading, and depending on the site of origin of this tumor, the criteria for pathological tumor staging differ as per the College of American Pathologists (CAP) protocol [3]. There are no defined criteria for grading uterine LMS, whereas three histological variants, including conventional, epithelioid, and myxoid LMS, were mentioned in the literature, with each having specific diagnostic criteria [4]. To confirm the smooth muscle origin of these tumors, immunohistochemical (IHC) markers [2] such as smooth muscle actin (SMA), desmin, and high molecular weight caldesmon (h-caldesmon) (more specific) are available when there is a diagnostic difficulty, chiefly while dealing with poorly differentiated and numerous histologic variants of LMS, although the expression may be weak or patchy in these scenarios. 

This study aims to analyze the demographic and clinicopathological profile of LMS cases from various anatomical sites that were diagnosed in our institution over a period of seven years, which includes infrequent presentation of these cases at unique anatomical locations, since it serves as one of the significant prognostic indicators of this tumor. 

## Materials and methods

Aim

The study aims at analyzing the demographic and clinical profile of LMS from various anatomical sites, which serves as one of the principal indicators of prognosis along with the histomorphological pattern.

Study design

This was a retrospective study of histopathologically proven LMS cases from January 2017 to December 2023 in the Department of Pathology at Sri Ramachandra Institute of Higher Education and Research, a tertiary care hospital in Chennai, India.

Study population

This study was done only on biopsied and/or surgically resected and pathologically proven LMS cases during the study period in the Department of Pathology of our hospital.

Inclusion Criteria

The study included all biopsied and/or resected specimens from various anatomical sites that were histopathologically diagnosed as LMS. Also included were the referral tissue slides and/or blocks that were received in the Department of Pathology for a second opinion, which were proven to be LMS.

Exclusion Criteria

Spindle cell neoplasms that were not proven as definitive diagnoses of LMS by histology and/or IHC were excluded from the study.

Data collection

Information gathering was done using the available medical records derived from the laboratory information system (LIS) at our institution. Histopathological data were collected from pathology case files from the Department of Pathology in our institution. The parameters that were studied include age, sex, anatomical site and size of the tumor, histological grade (FNCLCC grade for soft tissue LMS, based on tumor differentiation, mitotic count, and percentage of necrosis), pathological tumor, node, metastasis (TNM) stage, and IHC expression of these neoplastic cells by SMA and Ki-67 proliferation index.

Data analysis

Clinicopathological details were entered into Microsoft Excel software (Microsoft Corp., Redmond, WA) and analyzed. Descriptive statistics (frequencies, percentages, and means) were used to summarize the data.

Ethical approval

Approval was obtained in October 2024 from the institutional research ethics committee (IEC) of Sri Ramachandra Institute of Higher Education and Research (IEC reference ID CSP-MED/24/OCT/110/349) before the commencement of the study.

## Results

**Figure 1:**
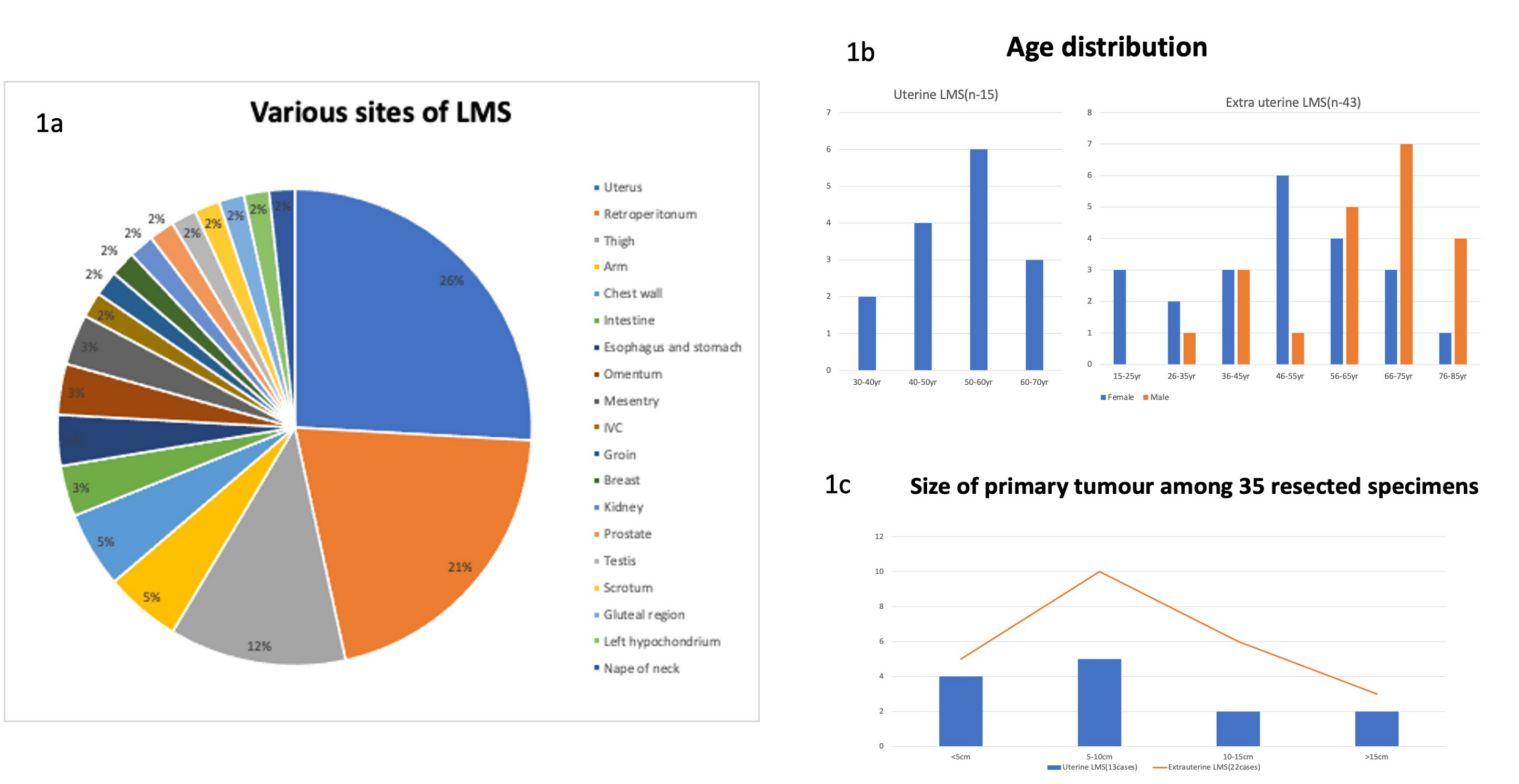
Clinical and demographic distribution of LMS 1a. The pie chart shows the various anatomical locations of primary LMS in our study expressed in percentage, with the uterus being the most common site. 1b. The bar diagram shows the age and sex distribution of uterine (n=15) and extrauterine (n=43) cases among 58 primary LMS in our study. 1c. The line column chart shows the size range of uterine (n=13) and extrauterine (n=22) LMS among 35 primary resected tumors. LMS: leiomyosarcoma; IVC: inferior vena cava

Out of 80 cases analyzed in this study, 45 (56.25%) were resected specimens, 25 (31.25%) were biopsies, and 10 (12.5%) were referral cases. Among the 80 cases analyzed, 58 (72.5%) were primary LMS, 17 (21.25%) were metastases from various sites, and five (6.25%) were recurrent lesions. From the 58 primary cases studied, 35 (60%) were resected specimens, and 23 (40%) were small biopsy specimens. As depicted in Figure 1a, the most common site of origin among 58 primary LMS cases in our study was the uterus, which comprised 26% (n=15) of the cases, followed by the retroperitoneum with 21% (n=12) and the thigh with 12% (n=7) of cases. Infrequent sites included the omentum, prostate, kidney, and testis, each constituting about 2% (n=1). Also, our study witnessed the incidence of LMS cases slightly more common among female patients than male patients, with a male:female ratio of 1:1.7, and the mean age of presentation was 55 years (Figure 1b). Figure 1c reveals the average size of the excised primary uterine tumors (n = 13) and extrauterine tumors (n = 22), which ranged from 2.5 cm to 25 cm and 1.6 cm to 30 cm, respectively. 

**Figure 2:**
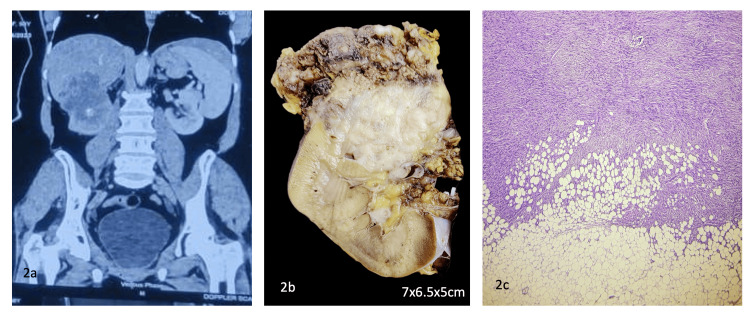
Rare presentation of primary renal LMS 2a. A CT scan of the abdomen showing a poorly defined solid mass involving the superior and interpolar region of the right kidney. 2b. A gross image showing the cut surface of the right nephrectomy specimen with a grey-white, solid, firm mass in the superior pole involving the perinephric fat. 2c. Microscopic section from the lesion showing fascicles and bundles of spindle-shaped cells with moderate atypia and extending into the perinephric fat. LMS: leiomyosarcoma

Figure 2 shows one of the examples of a rare presentation of a primary renal LMS case, initially presented with a complaint of right-sided abdominal pain for one week with no history of urinary disturbances, fever, or weight loss, and no significant past medical or surgical history. The general physical and systemic examination was also normal, with a soft, non-tender abdomen. Radiology revealed an exophytic, poorly defined solid mass measuring 7.6x7x6.5cm in the superior and interpolar region of the right kidney, suspicious of primary renal cell carcinoma (RCC), for which the patient underwent a right radical nephrectomy, and the histopathological examination revealed the final diagnosis of LMS. 

**Figure 3:**
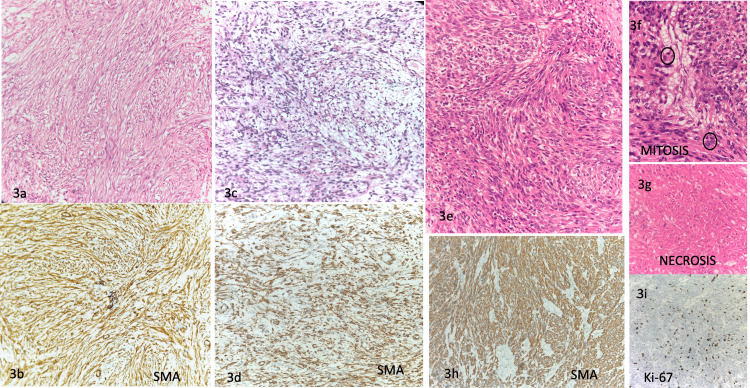
Histomorphological spectrum of LMS cases 3a. Sections from the arm lesion showing well-differentiated fascicles and bundles of spindle-shaped smooth muscle cells, with positive SMA-IHC (3b). 3c. Sections from the omental nodule depict the poorly differentiated morphology of tumor cells, which again showed positivity for immunohistochemistry staining of SMA (3d). 3e. Sections from uterine lesion showing fascicles and bundles of spindle-shaped cells exhibiting moderate atypia with increased mitosis (3f) and areas of necrosis (3g), which were positive for IHC–SMA (3h) and a Ki-67 labeling index of around 17% (3i). LMS: leiomyosarcoma; IHC: immunohistochemistry; SMA: smooth muscle actin

The microscopic examination of these LMS cases predominantly exhibited spindle cell morphology with fascicular arrangement. Only very few cases showed epithelioid morphology, which was quite challenging to diagnose without the aid of ancillary techniques. The histomorphological spectrum of presentation of LMS cases, irrespective of the site of origin, encompassed well-to-moderately differentiated and poorly differentiated LMS with or without necrosis in addition to increased mitotic rate (Figure 3). The three above-mentioned parameters were used to assess the grade of extrauterine soft tissue sarcomas based on the FNCLCC grading system, whereas the International Federation of Gynecology and Obstetrics (FIGO) and TNM staging were used for uterine LMS. In our study, 16 out of 22 extrauterine cases (around 73%) presented with histological (FNCLCC) grade 2, and while considering the pathological TNM staging, 11 cases (50%) were stage T2, six cases (27%) were stage T3, three cases (14%) were stage T1, and two cases (9%) were stage T4. Among the primary uterine resected LMS (n:13), 11 cases (85%) were limited to the uterus with pathological stage T1, whereas two cases infiltrating the abdominal soft tissue (stage T3) were noted. Almost all the cases tested with IHC for SMA and desmin showed positivity, along with a median Ki-67 labeling index of 30%. 

The metastatic lesions were found mainly in the liver, lung, and soft tissues and rarely in bone, which is detailed in Table 1 with their site of primary tumor origin. Among 17 metastatic cases, 12 (70%) were from the uterus, three (18%) from the retroperitoneum, and one (6%) each from the IVC and anterior chest wall. The liver is the most common metastatic site from uterine and retroperitoneal primary LMS, followed by the lung. Our study observed one uterine LMS showing metastasis to a rare site of the mediastinum. In addition to that, five recurrent lesions at the same site of resection were identified, two from the chest wall and one each from the uterus, forearm, and thigh, after a duration of around two to five years of primary diagnosis.

**Table 1:** Metastatic LMS cases (n=17) with their site of origin and the secondary deposit LMS: leiomyosarcoma; IVC: inferior vena cava

Primary tumor location	Number of cases (n=17)	Metastatic site
Uterus (n=12, 70.6%)	4 (23.5%)	Liver
	3 (17.7%)	Lung
	2 (11.7%)	Femur
	1 (5.9%)	Humerus
	1 (5.9%)	Mediastinum
	1 (5.9%)	Pelvis
Retroperitoneum (n=3, 17.6%)	2 (11.7%)	Liver
	1 (5.9%)	Lung
IVC (n=1, 5.9%)	1 (5.9%)	Thigh
Anterior chest wall (n=1, 5.9%)	1 (5.9%)	Lung

## Discussion

Soft tissue tumors are usually very challenging to diagnose only by histomorphological findings, as the spectrum of spindle cell lesions has a list of differential diagnoses, especially when they present at unusual sites. They are categorized based on their line of differentiation, according to the adult tissue they resemble, but it is not as straightforward as we assume, because when these tumors undergo malignant transformation, they may lose their line of differentiation. It is when we rely on the help of lineage-specific IHC markers to aid in an accurate diagnosis. For defining the smooth muscle origin, the key IHC markers include SMA, desmin, H-caldesmon, calponin, and myosin heavy chain (SM-MHC) [2]. In our institution, we use SMA and desmin to substantiate the smooth muscle origin of the spindle cell neoplasm we encounter. In this study, we only included the cases that were positive for SMA and desmin, with the confirmed diagnosis of LMS.

LMS generally occurs in the older age group (fifth to seventh decade of life) with a predilection more towards females, as witnessed in our study, even though the study cohort ranged from 15 years to 84 years. In addition, extrauterine LMS has a wide age group (from the second to the ninth decade) as compared to uterine tumors (from the fourth to the seventh decade). It is stated in the literature that hormonal influence and genetic factors play an essential role in the pathogenesis of LMS, which points towards the slight female predominance [5-7]. In females, the most frequent site is the uterus, which has abundant smooth muscle and is vulnerable to hormonal impact by estrogen and progesterone in addition to the genetic alterations that are associated with uterine LMS, including loss of PTEN, RB1, TP53, and ATRX along with MED12 mutation, PGR gene rearrangement, and PLAG1 gene fusion [8-11]. The most common non-uterine site is the retroperitoneum, followed by the gastrointestinal tract and extremities. These tumors exhibit additional mutations or pathway alterations involving PI3K/AKT/mTOR or MAPK signaling, DDR, and WNT/beta-catenin pathway genes with more pronounced chromosomal instability [9-11].

Our study observed the uterus as the most common site, ranking top on the list with 26%, followed by the retroperitoneum at 21%, extremities at 17%, and subsequently the gastrointestinal tract at 6%, and later on rare sites, each constituting 2% [12,13]. The infrequent sites of LMS included the kidney, testis, scrotum, prostate, omentum, breast, and groin region. There are many case reports publishing the incidence of LMS at unique anatomical sites [14-19]. This study compiles all the LMS cases that are diagnosed in our institute over seven years. A few examples of such odd incidences include the kidney, testis, prostate, breast, and mesentery. The probable cell of origin of LMS at these infrequent sites could be explained by the smooth muscle cells derived from the blood vessels supplying those organs and/or tissues.

The substantial tumor sizes documented in this study included 24.5 cm, 26 cm, and 30 cm from the uterus, thigh, and retroperitoneum, respectively, which further states that extrauterine LMS tends to be larger than uterine LMS. Grossly, these tumors give a fleshy, grey-white to tan and whorled appearance irrespective of their site of origin, which is a distinct macroscopic feature of LMS with or without grey-black friable areas that mark the hemorrhagic and necrotic areas, pointing towards a higher grade of the tumor. Microscopically, both soft tissue and uterine LMS depict a similar spindle cell morphology with individual cells showing moderate to abundant, pale to bright eosinophilic cytoplasm with plump, blunt-ended nuclei exhibiting varying degrees of nuclear pleomorphism, mitosis, and necrosis, based on which LMS can be graded according to FNCLCC grading. 75% of extrauterine LMS presented with FNCLCC grade 2, the rest with grade 3 (18%) and grade 1 (7%) in our study.

The major histopathologic subtypes include epithelioid LMS and myxoid LMS, next to the conventional LMS, and very rare entities include inflammatory LMS [20], pleomorphic LMS, and dedifferentiated LMS, which were not encountered in our study. Regardless of the histologic variants, pathological tumor staging for LMS is based mainly on the site, size, and extent of involvement. Regarding the uterine LMS in our study, all of them were limited to the uterus, which falls under the pT1 category, whereas most extrauterine LMS fall between pT2 and pT3. No nodal or distant metastatic involvement was identified at the time of primary diagnosis, whereas 17 metastatic lesions were documented in this study; among them, only three cases were primarily diagnosed in our institute, with a time interval of 3.4 to 6.5 years. For the remaining metastatic lesions, the primary was diagnosed elsewhere, and the patient presented to us only for the metastatic lesions, with proper evidence of LMS diagnosis. One uterine LMS showed recurrence at the site of the posterior wall of the urinary bladder. Two chest wall LMS and one forearm and one thigh lesion showed recurrence at the same site with a time range of two to five years.

Limitations and future prospects

This study had a few limitations. This was a single-center study. Multi-institutional data collection would provide valuable insights into the precise nature of the disease. Clinical follow-up of these cases and prognosis may be assessed to design the appropriate management protocol and optimize the patient outcome pertaining to the site of origin, thereby enhancing the effectiveness and efficacy of healthcare delivery.

Further molecular workup can be done to understand the exact pathogenesis behind the uterine and extrauterine tumors and to comprehend the similarities and variations between these associations, which would provide valuable insights into patient management. 

## Conclusions

The results of our study witnessed the plethora of LMS cases at diverse anatomical locations with varying grades of presentation. The overall prognosis varies with respect to the location, grade, and stage of the tumor. Hence, the comprehensive assessment, keeping in mind the primary location of these tumors, is recommended as they have varied biological behavior. However, further molecular evaluation of these tumors may be warranted to understand the possible course of the disease and overall outcome.
